# A Unifying Theory for SIDS

**DOI:** 10.1155/2009/368270

**Published:** 2009-10-29

**Authors:** David T. Mage, Maria Donner

**Affiliations:** ^1^A. I. duPont Hospital for Children, Biomolecular Core Laboratory, 1600 Rockland Road, Wilmington, DE 19803, USA; ^2^DuPont Haskell Global Centers for Health & Environmental Sciences, Investigative Sciences, Newark, DE 19711, USA

## Abstract

The Sudden Infant Death Syndrome (SIDS) has four distinctive characteristics that must be explained by any theory proposed for it. (1) A characteristic male fraction of approximately 0.61 for all postneonatal SIDS in the US; (2) a distinctive lognormal-type age distribution arising from zero at birth, mode at about 2 months, median at about 3 months, and an exponential decrease with age going towards zero beyond one year; (3) a marked decrease in SIDS rate from the discovery that changing the recommended infant sleep position from prone to supine reduced the rate of SIDS, but it did not change the form of the age or gender distributions cited above; (4) a seasonal variation, maximal in winter and minimal in summer, that implies subsets of SIDS displaying evidence of seasonal low-grade respiratory infection and nonseasonal neurological prematurity. A quadruple-risk model is presented that fits these conditions but requires confirmatory testing by finding a dominant X-linked allele protective against cerebral anoxia that is missing in SIDS.

## 1. Introduction

The Sudden Infant Death Syndrome (SIDS) was defined in 1969 [1]. Since then, more than 9000 articles related to SIDS were published and over 100 SIDS explanations appeared in *Medical Hypotheses* [2]. SIDS (9ICD 798.0; 10ICD R95) is a diagnosis of exclusion. SIDS occurs when an infant dies suddenly, unexpected by history, and without a cause found at forensic autopsy or thorough death-scene investigation. In the past four decades several multifactor models have been proposed to explain the SIDS phenomenon [3]. For example, Emery [4] proposed a “three inter-related causal spheres of influence model” in which any two of these three could cause SIDS: (1) subclinical tissue damage (2) deficiency in postnatal development of reflexes and responses, and (3) environmental factors. Filiano and Kinney [5] also proposed a “triple risk model” but required three risk factors, similar to Emery's, to act simultaneously and described them as (1) vulnerable infant; (2) critical development period; and (3) environmental stressors. These and other models do not, however, address the mathematical character of SIDS. This paper addresses each characteristic factor of SIDS that must be explained: the gender distribution; the age distribution; the effect of prone and supine sleep positions; the seasonal variation; risk factors of anemic apnea, respiratory infection, and neurological prematurity, and links them together with other causes of respiratory deaths as a proposed unifying theory.

## 2. Materials and Methods

This paper uses data on infant live births and deaths as reported by the U.S. Centers for Disease Control and Prevention (CDC) [6]. The SIDS data are given by International Classification of Diseases (ICD) for 1979–1998 as 9ICD and for 1999–2005 as 10ICD. Although technically a SIDS diagnosis requires an autopsy without causal findings, not all these SIDS were autopsied and the percentage of SIDS without autopsy in the US has decreased monotonically from 1979 to the present day. However, CDC lists SIDS for all autopsied and nonautopsied cases without distinction. In the case of an interracial parentage, CDC only reports a single race as usually chosen by the mother. Consequently the cause of death and race of SIDS will have measurement error involved which will increase the chi-square 1 d.f. test statistics (*χ*_1_^2^) of comparisons between predicted and observed SIDS numbers and races, above those for tabulated *P*-values that assume the variance is from sampling error only. Therefore although *P*-values are presented they may give incorrect implications for rejection of hypotheses if they fall < *P* = .05. We therefore rely upon the overall consistency of age and gender data to support our mathematical construct of SIDS.

## 3. Results

### 3.1. The Gender Distribution of SIDS

**Figure 1:**
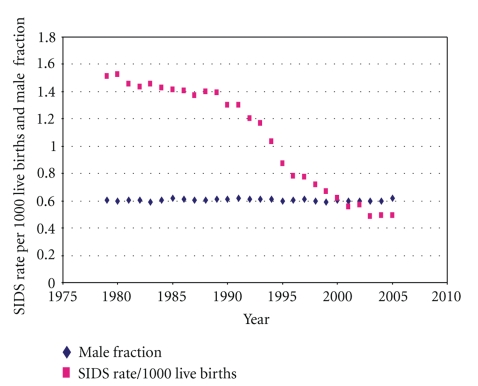
US postneonatal SIDS during period of change from prone to supine sleep position showing that the male fraction remains constant at or about 0.61.

A characteristic male fraction is associated with SIDS. Because of difficulty in detecting congenital anomalies and other subtle causes of death in <28-day neonates, postneonatal SIDS are usually studied to avoid false positives [3]. The CDC [6] reports there were 62,933 male and 40,952 female postneonatal SIDS during 1979 to 2005 for a male fraction of 0.606. Figure 1 shows the male fraction of US postneonatal SIDS of all races over this period fluctuating slightly about the mean value of 0.606 as the SIDS rate decreased markedly from the discovery that the prone sleep position was a major SIDS risk factor which was followed by a back-to-sleep campaign in 1992.

Naeye et al. [7] first hypothesized that the male excess in infant mortality could be X-linked. Mage and Donner [8–10] showed that this excessive male fraction could be related to an unknown X-linked gene locus with a dominant allele (*A*) protective against SIDS, perhaps by providing enzymatic activity to allow anaerobic oxidation to take place in respiratory control neurons of the brainstem during transient periods of cerebral anoxia. The corresponding recessive allele (*a*), with frequency *q*, would not provide this protection and could allow critical cerebral neurons to die of anoxia, and SIDS to occur when the dominant allele *A* is not present.

For XY males and XX females the recessive allele frequency (*q*) can be determined from (1a) and (1b), as the ratios of susceptible infants

(1a) $$\begin{array}{c} \frac{\text{Fm}}{\text{Mm}}=\frac{q^{2}\text{Fb}}{q\;\text{Mb}}, \end{array}$$

(1b) $$\begin{array}{c} q=\frac{\text{Fm}/\text{Fb}}{\text{Mm}/\text{Mb}}, \end{array}$$

where Fm and Mm are postneonatal female and male SIDS and Fb and Mb are female and male live birth rates, respectively, so *q* represents the female postneonatal SIDS rate divided by the male postneonatal SIDS rate per 1000 live births.

The global average male fraction of 0.612 for *autopsied* postneonatal SIDS is higher than the 0.606 US male fraction for total autopsied and nonautopsied SIDS, perhaps due to false positive SIDS that have a lower male fraction [8]. With a 5% average male excess live birth rate, 0.612 corresponds to an average recessive allele fraction of *q* = 2/3 [8]. When stratified by race, we obtain from the CDC US 1979–2005 total postneonatal SIDS and birth data there is a White male fraction of 0.622, Black male fraction of 0.570, and other races combined = 0.594 [6]. Using (1b), *q* White = 0.639, *q* Black = 0.779, and for other races combined *q* = 0.724. Such variation in allele fraction between races along with the establishment of Hardy-Weinberg equilibrium is expected for each racial grouping from genetic drift over a long period of time. By necessity we accept here the CDC [6] racial designations and neglect the presence of interracial infants.

### 3.2. Other Respiratory Diseases and Traumatic Events with the Same Male Fraction as SIDS

In our genetic analysis we assume that all SIDS infants have only the recessive allele (*a*) and require their probability of genetic susceptibility Pg to equal 1. To support this genetic mechanism, we note in Table 1 that other causes of respiratory deaths in infancy have a statistically similar male fraction to 0.606 for postneonatal US SIDS when all races are combined. Not all of these CDC [6] reported cases were autopsied, and false positive SIDS occur from infanticide by gentle suffocation that is virtually indistinguishable from SIDS at autopsy [11], so statistical testing assuming no autopsy error may not be cause for rejection at *P* = 0.05. 

RDS, also known as hyaline membrane disease, had a male fraction of 0.610. Bronchopulmonary Dysplasia had a male fraction of 0.613. With the discovery that the prone position is a risk factor, there is a trend to “parse” postneonatal SIDS into “true” SIDS and subcategories [12]. Two such alternatives to SIDS are Accidental Suffocation and Strangulation in Bed and Unknown Ill-defined Unspecified-Causes which had male fractions of 0.593 and 0.597, respectively. Acute Upper and Lower Respiratory Infections had a male fraction of 0.619. Suffocations by Inhalation of Food or Other Foreign Object (SIFFO) in infancy had a male fraction of 0.600 very close to 0.606 (*P* = 0.52) for all postneonatal SIDS [6]. The risk factors for infant inhalation of food or other object are morsel size, rounded shape, and slippery surface, like a grape [13]. However, types of infant food, and mode and manner of preparation are identical for males and females, so these risk factors are independent of gender. We hold that all this tabulated male fraction similarity of order 0.61 is strong evidence of a common X-linked recessive susceptibility to the same terminal mechanism of cerebral anoxia.

Furthermore, the *virtually identical* male fraction of 0.6053 compared to 0.6057 for SIDS occurs for these same SIFFO ICD codes combined for all children ages 1 to 14 years in the US from 1979 to 2005, with 2,324 male and 1,515 female (*P* = 0.98). When broken into ages 1–4, 5–9, and 10–14 years none of these groups are rejected [6]. The implications of this consistent male fraction from infancy through adolescence is emphasized in the later discussion section. The SIFFO + IGC data for the next CDC age group of 15–19 years with a higher male fraction is not shown here because higher teenage male alcohol consumption is a new positive bias factor (496 male, 277 female: male fraction = 0.642).

The male fractions in 1979–1998 of all US infant deaths by all ICD 9 Chapters and for 1999–2005 in their ICD 10 equivalents are shown in Table 2. These data show the well-known male excess in virtually all ICD classes of infant death, with only the neoplasms showing no male or female excess as expected from a purely random initiation process as the 5% US male live birth excess corresponds to a male fraction of 105/205 = 0.5122. Two important observations can be made.

(1) The differing male fractions for most of these disease classes are essentially similar between the two periods 1979–1998 and 1999–2005. This suggests that there is something physiological involved that provides the apparent characteristic excess male risk for each such class of cause of death. For example, certain conditions arising in the perinatal period with some 350 000 deaths covered by ICD9 and 100 000 covered by ICD10 have male fractions of 0.566 and 0.567, respectively.

(2) The approximately 0.61 male fractions of Table 1 for respiratory causes shown are found as expected for the congenital anomalies of the respiratory system (0.602 ICD9 and 0.579 ICD10) and diseases of the respiratory system (0.587 ICD9 and 0.581 ICD10). What is surprising is that the male fraction of mortality from diseases of the digestive system in infancy is similar to that from respiratory causes, 0.588 in 9ICD and 0.601 in 10ICD. In the UK from 1979 to 2006 “Other diseases of the digestive system” (not ulcer-appendicitis-hernia-obstruction-chronic liver disease-cirrhosis) were 458 male and 329 female for a male fraction of 0.581 similar to the US data [14]. We speculate that a linkage between the mechanism for the similar male fraction from digestive disease as SIDS may be from digestive causes such as malabsorption of iron and glucose in celiac disease and insufficient vascularization that would limit uptake and transport of glucose, respectively. This could lead to hypoglycemia that is a known risk factor for SIDS and sudden death [15, 16]. “In the older infant, the resistance to hypoxia is much less than for the neonate, reflecting the diminished stores of glycogen and therefore limited substrate for anaerobic metabolism [3].”

An enzyme, such as Glucose-6-phosphate dehydrogenase (G6PD) could play a role [15] as its X-linked gene locus is at Xq28 and it has a great multiplicity of alleles that are associated in their deficiency with nonspherocytic hemolytic anemia [17], and anemia is a likely risk factor for SIDS [18]. G6PD catalyzes initiation of glucose oxidation via the hexose-monophosphate pathway that may be a critical requirement for neuronal survival during cerebral anoxia.

There could be more complicated X-linked processes such as requiring two (or more) independent X-linked alleles with probabilities *q*1 and *q*2, with probability of simultaneous presence (*q* = *q*1*q*2) that would equal the *q*values listed above for a single X-linked allele. Alternatively, a gene locus such as G6PD could have many recessive alleles (*q*1, *q*2, *q*3,…) that are nonprotective of SIDS that could sum up to the *q* values listed above for the same risk of SIDS (*q* = *q*1 + *q*2 + *q*3 + ⋯).

We have chosen a single-gene X-linkage process for simplicity of discussion, and note that any genome-wide association study required to test our model can test for all possibilities.

### 3.3. The Age Distribution of SIDS

**Figure 2:**
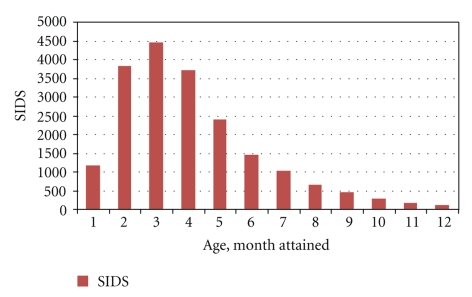
Age distribution from 15 global data sets combined versus month attained of 19,949 SIDS [21].

The age distribution of SIDS is unique: “Any viable hypothesis for the cause of SIDS must account for its characteristic age distribution.” [19]. Raring [20] first noted that the unique and characteristic age distribution of SIDS appeared to follow a 2-parameter lognormal model. Mage [21] reviewed the SIDS age literature and in a meta-analysis of 15 global SIDS age data sets obtained the distribution of some 20 000 ages of SIDS shown in Figure 2. In construction of Figure 2, 1-month is <28 days of life. Other monthly intervals are approximate as 365 is not divisible by 12. Age data in weeks of life were divided by 4.33 to convert to months and the Althoff [22] data from Cologne reported as age within midmonth intervals (e.g., 1.5–2.5 month) were plotted to estimate the corresponding integer month intervals (e.g., 1-2 months and 2-3 months) for pooling with the other monthly SIDS data. The total of 19,949 SIDS includes 194 SIDS deaths that are predicted to occur after 1-year in these 15 cohorts by use of an exponential fit to monthly data intervals 5 to 12 that was then extrapolated and summed from 13 to 41 months.

These data in Figure 2 were fit by a 4-parameter lognormal distribution, also known as the Johnson S_B_ distribution [23], shown as (2). Here dp(*m*) is the probability of SIDS occurring between ages *m* and *m* + dm in months, median *μ* = 3.1 months and standard deviation *σ* = 0.6617, as fit by maximum likelihood [21]

(2) $$\begin{array}{cc} & \frac{\text{dp}\left(m\right)}{\text{dm}}=\sqrt{{\left(2\pi \sigma^{2}\right)}^{-1}}\left[{\left(m+0.31\right)}^{-1}\;+\;{\left(41.2-m\right)}^{-1}\right]\\ & \;\times \exp\;\;\left[-{{\log\;}_{\text{e}}}^{2}\left(\left[\left(m+.31\right)\left(41.2-\mu \right)\right]/\left[\left(41.2-m\right)\left(\mu +.31\right)\right]\right)/2\sigma^{2}\right]. \end{array}$$

Equation (2) can be interpreted as a sum of products of three age dependent terms, denoted as Pn, Pi, and Pa.

### 3.4. Pn, Risk of Neurological Prematurity

Let Pn = 1/(*m* + 0.31) represent a risk factor of neurological prematurity leading to delays in development of respiratory reflexes and responses, that decreases with increasing age.

Neurological prematurity is a risk factor that is maximal at birth and decreases as the infant physically matures. Kinney [24] has found that an important subset of SIDS appears to have a deficiency in serotonin receptors that is hypothesized as a causal factor of those SIDS.

### 3.5. Pi, Probability of a Low-Grade Respiratory Infection

Let Pi = 1/(41.2 − *m*) represent an infection risk factor that increases with increasing age.

A low-grade respiratory infection is a risk factor for SIDS. Emery and Weatherall [25] and Øyen et al. [26] discuss a class of infant deaths, sometimes called “secondary SIDS,” that have findings of low-grade respiratory infection at autopsy that of itself is insufficient to cause death. Risk of such infection increases with age as infants lose passively acquired maternal immunoglobulin (IgG) and they have increased exposure to pathogens as they have more contacts both within and without their immediate family.

US DHHS [27] linked birth and death certificate data for 1995–2004 show, in Table 3, that the rate of SIDS increases monotonically with live birth order (LBO). It has been suggested that older school-age siblings may be an important respiratory infection vector [3]. We assume here that the infant lives with two parents, all older siblings survived to the time of SIDS death, and no adoption of the SIDS infant or older siblings took place. For LBO ≥6 we assume only 5 siblings have contact with the infant.

Let the probability of a family member *not *carrying a respiratory infection communicable to the infant at any time = **P**. For infants with family size = 2 parents + (LBO −1) siblings the probability of not having an infection vector present is equal **P** ^(LBO+1)^. The probability of an exposure to at least one carrier is then 1 − *P* ^(LBO+1)^. By least squares analysis we found *P* = 0.9 with a scaling factor of 2.5, so we model the rate of SIDS per 1000 = 2.5∗ (1. − 0.9^(LBO+*  *1)^) and show our predictions in the lower row in Table 3. The unweighted correlation of predictions and observations is *r* = 0.9966.

### 3.6. Pa, Probability of Physiological Anemia Causing Apnea and Hypoxia that Are SIDS Risk Factors

Infant anemia has not been considered directly as a risk factor for SIDS *per se*, because “accurate hemoglobin [Hb] levels cannot be determined after death [18]” due to rapid Hb breakdown resulting in the mottled and reddened areas known as *livor mortis*. A study in mice shows how Hb is already significantly decreased in the first postmortem hour [28]. Because the exact time of SIDS during sleep is not known it would be impossible to correct for the variable amount of Hb lost between the instant of SIDS death and autopsy. “There is, however, indirect evidence suggesting a relationship between anemia and SIDS: the peak incidence of SIDS coincides with the nadir [of Hb] in the physiological anemia of infancy.” [18].

Anemia does contribute to apnea and apparent life-threatening events (ALTEs) from causing longer cyanotic breath-holding spells [29–32] that are risk factors for SIDS, leading to “The Apnea Hypothesis.” [3, 32]. Therefore anemia is treated by us as a risk factor for SIDS.

Let Pa = exp [−log _*e*_^2^ ([(*m* + 0.31)/(*μ* + 0.31)]/[(41.2 − *m*)/(41.2 − *μ*)])/(2*σ* ^2^)], as found in the Johnson S_B_ model [23] as (2), represent an anemia-*cum*-apnea risk factor rising from 0 at birth, reaching a peak at the median (*μ* = 3.1 months) and decreasing to zero at 41.2 months.

Anemia in infancy may be defined relatively as any value for the hemoglobin [Hb] less than two standard deviations (<–2*σ*) below the mean for age [33], or absolutely as less than a fixed value, such as 13.5 g/dL which is the −2*σ* level below mean cord blood Hb and mean Hb at 1 week [34]. Infant physiological anemia is a risk factor that is virtually zero at birth due to placental transfusion during labor [35] and at birth Hb concentration in the blood can reach +2*σ* of 23.7 g/dL [36]. We propose that this high at birth Hb phenomenon accounts for the relative protection from SIDS during the first week of life. In the following weeks, total Hb decreases rapidly as fetal hemoglobin (HbF) is removed faster than it can be replaced by adult hemoglobin (HbA). A nadir in total Hb occurs at or about 2 months of age for a term infant that corresponds to the 63rd day mode of the SIDS S_B_ age distribution [18, 21]. Table 4 shows the −2*σ* Hb g/dL level (lowest 2.5% of all infants) [33]. 

By definition, approximately 25 in 1000 term infants have a Hb value below the −2*σ* value shown, and preterm infants will fall under this value with a higher frequency, perhaps related to their increased risk of SIDS. Of those 25 in 1000, the one with the lowest Hb would be at the highest risk of apnea and therefore SIDS.

If physiological anemia is considered as a Hb deficit from a fixed level of 13.5 g/dL, that could, combined with apnea, cause transient hypoxia and inability to meet neuronal oxygen demand in the brainstem of SIDS susceptible infants [18]. If so, it could correspond, as shown in Table 4, to the rise-and-fall factor Pa modeled from the JohnsonS_B_distribution as (2) fit to Figure 2.

### 3.7. Seasonal Variation of SIDS Rate with a Winter Maximum

The presence of respiratory infection as a risk factor fits the characteristic of SIDS of a seasonal dependency, maximizing in the winter and minimizing in the summer, that has been associated with wide seasonal temperature changes [3]. Mage [37] showed that in Hawaii, a semitropical US state with only narrow seasonal change in mild temperatures, that 384 SIDS varied seasonally with calendar day (*t*) between 1979 and 2002 as a cosine function shown as (3) where the maximum SIDS rate is predicted to occur on January 30th (*t* = 30):

(3) $$\begin{array}{cc} \text{Mortality}\;\text{on}\;\text{Day}\;t=0.810+0.241\;\left[1+\text{cosine}\frac{2\pi \left(t-30\right)}{365.25}\right], & \\ 0<t<365.25. & \end{array}$$

This equation may be interpreted to show that in Hawaii, 23% of SIDS have a seasonal infection component (0.241/1.051) and that 77% of SIDS occur at a constant rate due to other physiological factors such as anemia and neurological prematurity that have no known seasonality. The winter flux of infection vector would come via visitors from the US mainland and Japan, so the authors expect that the proportion of seasonal infections and SIDS may be larger in temperate zones of the US with colder winter temperatures than found in Hawaii.

There has been a tendency for the winter peak to be reduced since the start of the back-to-sleep campaign that may be due to the lessening of the hypoxia caused by low-grade seasonal respiratory infection when sleeping supine [38, 39].

### 3.8. Similar Age Distribution for Prone SIDS and Supine SIDS

**Figure 3:**
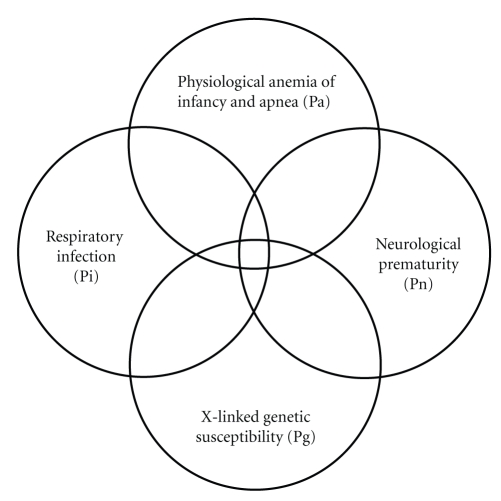
Venn Diagram for a Quadruple Risk Model of SIDS. These four probability factors involved with SIDS explain the age and gender distributions invariant with different sleep position, and subsets of SIDS found with and without neurological prematurity and respiratory infection. It is proposed that a prone infant is susceptible to SIDS anywhere in the intersection between the genetic (Pg) and anemia-related apnea (Pa) factors, but a supine sleeping infant is only susceptible to SIDS if it is in the intersection of all four factors (Pa, Pg, Pi, Pn).

The pre-1992 lognormal form of the age distribution of SIDS [40, 41] remained the same during the change of preferred sleep position from prone to supine. Pollack [40] found the age distributions of US SIDS between 1989 and 1999 were virtually unchanged in the two cohorts. He reported that “the stability of this distribution is remarkable when one considers the large decline in SIDS incidence”—as shown in Figure 1. Malloy and Freeman [41] also found little change in age distribution for US SIDS between 1992 and 1999 (*P* = 0.025). The derivation and its explanation for this consistency is aided by a Venn Diagram shown as Figure 3. 

Let a prone sleeping infant be susceptible to SIDS in both the Pg Pa Pn and Pg Pa Pi areas of Figure 3 even if missing the Pi or Pn risk factors, respectively. Note that the sum of Pg Pa Pn + Pg Pa Pi represents two overlapping areas on the Venn diagram because the central segment (Pg Pa Pn Pi) is counted twice. Let a supine infant be susceptible to SIDS only in that central segment.

Let the probability of a prone sleeping child = Pp and that of a supine sleeping child = Ps. For simplicity we include the side sleeping position with the prone, and we require Pp + Ps = 1. Then the probability of dying of SIDS at age m while prone (Ppsids) is written as,

(4) Ppsids=Pp  Pg  Pa  ([Pn+Pi]−PnPi).

One can then write the probability of supine SIDS (Pssids) as,

(5) Pssids=Ps  Pg  Pa  Pn  Pi.

Combining (4) and (5) we get the total probability of SIDS (Psids) as

(6) Psids=Pp  Pg  Pa  (Pn+Pi)+(Ps−Pp)  Pg  Pa  Pn  Pi.

We then note that the sum of Pn + Pi has a similar mathematical form as Pn Pi as follows:

(7a) $$\begin{array}{cc} \text{Pn}+\text{Pi} & =\frac{1}{\left(m\;+\;0.31\right)}+\frac{1}{\left(41.2-m\right)}\\ & =\frac{\left[\left(41.2-m\right)+\left(m+0.31\right)\right]}{\left[\left(m+0.31\right)\;\left(41.2-m\right)\right]}, \end{array}$$

(7b) $$\begin{array}{cc} \text{Pn}+\text{Pi} & =\frac{41.5}{\left[\left(m+0.31\right)\left(41.2-m\right)\right]} \end{array},$$

(7c) $$\begin{array}{cc} \text{Pn}\;\text{Pi} & =\frac{1}{\left[\left(m+0.31\right)\left(41.2-m\right)\right]}. \end{array}$$

Thus the mathematical form for the age distribution of both supine SIDS and prone SIDS can be represented by the same relationship of C Pa/[(*m* + 0.31) (41.2 − *m*)], where C is a constant, which implies that, in terms of relative probability at different values of *m*, (7b) and (7c) are the same. This is consistent with the report that there were similar frequencies of pathological findings in both supine and prone SIDS confirming that the mode and cause of SIDS death is apparently the same for both sleep positions [42].

This derivation shows how the Venn Diagram and Johnson*  *S_B_age distribution predict that supine and prone SIDS have the same age distribution, with lower rates for the supine SIDS. This corresponds to the supine requirement to have all 4 risk factors (Pg Pa Pn Pi) as opposed to only 3 risk factors (Pa Pg Pi or Pa Pg Pn) that can allow a prone SIDS to happen more readily. Factors that make the prone sleep position a risk factor for SIDS are rebreathing of exhaled breath with reduced oxygen and increased carbon dioxide [43] and the finding that presence of a fan in the infants sleep environment, that disperses exhaled breath, decreases the SIDS rate [44].

## 4. Discussion

Other hypotheses than the X-linkage hypothesis of Naeye et al. [7] for the male excess in SIDS and other causes of infant respiratory mortality have appeared in the literature [45–47]. Finnström [48] reviewed this topic and concluded that “The mechanism behind the excess perimortality rate in male infants is not known. A genetic factor leading to reduced tolerance to hypoxia is possible.”

Torday et al. [45] analyzed amniotic fluid and showed the male fetus developed pulmonary surfactants slower than the female fetus and suggested that this deficit at birth may cause the male excess in infant respiratory distress syndrome (RDS) that matches that of SIDS. This is not likely because the measured deficit should decrease with maturity as the infant ages, but CDC reports that the male fraction of RDS between 28 and 364 days [0.617] is greater than the male fraction [0.604] on the first day of life when the deficit is maximal [6].

Patterson et al. [46] found in their SIDS cases that males had a larger deficiency in serotonin receptors in the brainstem than females and suggested that this may be related to the male excess in SIDS. As for the male surfactant deficit cited above, a greater male serotonin-receptor deficit at birth should decrease with infant maturity, but the 0.606 male fraction of SIDS between 28 and 364 days is also greater than the 0.548 male fraction for 0–6 days [6] (which may partially be related to false positive SIDS from undiscovered infanticide or subtle congenital anomalies).

L'Hoir et al. [47] found in their study in the Netherlands that male infants were placed to sleep in the prone position more often than females, and were more likely to turn prone from a side sleeping position than females, and suggested that this may be related to the male SIDS excess. However, as shown in Figure 1, the SIDS male fraction remained essentially the same as the recommended sleep position in the US changed from prone (pre-1992) to supine (post-1992), even though the SIDS rate dropped by a factor of three from 1979 to 2005 [6].

Furthermore, any other hypothesized cause for SIDS that suggests that the SIDS male excess in mortality is related to a male underdevelopment relative to the female cannot explain the fact that *virtually exactly* the same male fraction of 0.605 occurs for SIFFO between 1 and 14 years as the 0.600 in the first year of life shown in Table 1. The risk factors for SIFFO in children are independent of gender because food in the US is not chosen or prepared differently for males and females. Types of food that are most often recovered from the upper airway at infant autopsy are raw carrot and apple, round and slippery items such as hotdog pieces without skin removed, candy, nuts, and grapes [49–51]. Foreign objects swallowed by children over 1 year of age are often balloons and small coins such as pennies. Although the rates of SIFFO decrease with age, as dentition and swallowing control develop, and the types of food items eaten by children change as they go from infancy to 14 years of age (e.g., chewing gum is often inhaled), the male excess remains the same up to 14 years. As opposed to SIDS that predominantly occurs during sleep, SIFFO predominantly occurs while the infant is awake or being fed, and immediate first aid is attempted that is successful in approximately 99% of all cases [52, 53]. Yet, assuming equal SIFFO risks for males and females, more males than females cannot be resuscitated in exactly the same proportion as dying in SIDS. Virtually all other risk factors posited for SIDS are either independent of gender (e.g., parental smoking or autosomal genetic conditions) or are inoperative for SIFFO between 1 and 14 years of age—except the possible X-linkage.

An obvious potential cause of an infant male excess for any ICD class may be due to an androgen excess in the male. We discussed this previously [54] and showed that during the first year of life, the SIDS male fraction remains relatively constant while the male serum testosterone is slightly higher than the female's at birth, peaks for three months during the first six months to aid testicular descent, and falls back towards zero for the second six months of life, so an androgen interaction is not a likely factor.

As in any epidemiology study, there is always a finite probability that these data are the result of happenstance and coincidence, known as sampling error, and that next year's data may be cause for rejection of the developments presented. However, because of the large sample sizes analyzed this probability is virtually zero.

## 5. Conclusions

We have shown how all characteristic properties of SIDS, its gender, age, and seasonal distributions, along with the observed risk factors of apnea, respiratory infection, and neurological prematurity, can be tied to each other mathematically. These relationships presented here explain how the supine position reduces the rate of SIDS and why it does not change the gender distribution or the form of the age distribution from those of SIDS occurring predominantly in the prone position. Because all SIDS risk factors except the hypothesized X-linkage are independent of gender, we propose that equal numbers of males and females, per equal numbers of live births, are at risk of having potentially fatal risk factors that we previously defined here as Pa, Pi, and Pn. Approximately 2/3 of all males and 4/9 of all females have a genetic risk factor Pg that is necessary to cause SIDS—but not sufficient by itself—resulting in the fixed proportion of observed male and female death rates. Infants with the protective allele and the three other risk factors (see Figure 3) may be among the cohort of those presenting with apparent life-threatening episodes (ALTEs) that do not then or later progress to SIDS.

It is proposed that SIDS may occur for those genetically susceptible infants when repeated transient coincidences of factors reduce the oxygen supply (apnea, anemia, rebreathing exhaled breath, etc.) during a period of increased oxygen demand (low grade respiratory infection raising body temperature). If the infant has a residual neurological prematurity, auto resuscitation by the gasp reflex may be delayed causing acute cerebral anoxia that may cause some respiratory-drive neurons in the brainstem to die (Emery's “subclinical tissue damage” [4]). When a sufficient number of such neurons die, the next sleep with identical risk factors causing anoxia may reduce the number of functioning neurons below a minimum critical requirement so auto resuscitation is impossible. The protected infant with an X-linked dominant allele (*A*) could switch over from aerobic oxidation to anaerobic oxidation to keep those critical neurons alive during the same transient anoxic conditions so that autoresuscitation could occur.

In summary, the quadruple risk model presented here, with factors developed from pre-1994 gender data [8] and from pre-1992 SIDS age data [21], predicts the age and gender distributions for post-1992 data as shown. The factors determining the age distribution mesh with the medical literature's findings of the risk factors for SIDS. Should the genetically susceptible infant pass through infancy unscathed, the genetic susceptibility to cerebral anoxia can still penetrate in childhood if anoxic circumstances arise as shown by the identical US postneonatal SIDS male fraction of 0.606 occurring in US children aged 1 to 14-years suffocating from inhalation of food or other foreign objects [6]. So, in the absence of any other plausible explanation in the medical literature for the same SIFFO male excess from birth to 14 years of age as SIDS, a common X-linkage remains as the only possibility. Furthermore there was a 45% excess adult male completion rate of suicide attempts by coal-gas inhalation in Paris between 1949 and 1962 (completions of 58% male versus 40% female) [55].

In conclusion, although modern thought is now that SIDS is a composite of independent and different causes of death, they all appear to have the same male fraction. We reason that all those different causes of death lead to the same cerebral anoxia that may result in respiratory failure from the absence of an X-linked dominant allele that supports anaerobic oxidation in respiratory control neurons of the brainstem. Proof of this unifying mechanism must await genetic testing to identify, if correct, the unknown recessive X-linked allele that is exclusively present in all these ICD codes with the statistically similar male excess of SIDS.

## Figures and Tables

**Table 1:** US Mortality Data for 1979–2005 showing infant respiratory deaths with statistically similar male fraction as SIDS and sets of older child suffocation deaths by inhalation of food or foreign object having the statistically similar male fraction as 0.606 for SIDS [6].

Disease*	Age	ICD 9 Codes 1979–1998	ICD 10 Codes 1999–2005	Male death	Female death	Male fraction	Chi-square *P*- value for 1 d.f.
SIDS	28–364 days	798.0	R95	62,933	40,952	0.606	Reference
RDS	0–364	769	P22.0	39,990	25,590	0.610	0.036
BPD	0–364	770.7	P27.1	6,547	4,127	0.613	0.11
ASSB	0–364	913.0	W75	2,843	1,948	0.593	0.08
UNK	0–364	799.9	R99	9,931	6,695	0.597	0.025
AULRI	0–364	460–466	J00-J06	1,712	1,053	0.619	0.15
SIFFO+IGC	0–364	911, 912	W78-W80	2,035	1,356	0.600	0.52
SIFFO+IGC	1–4 years	911, 912	W78-W80	1,651	1,034	0.615	0.31
SIFFO+IGC	5–9	911, 912	W78-W80	371	272	0.577	0.14
SIFFO+IGC	10–14	911, 912	W78-W80	302	209	0.591	0.49
SIFFO+IGC	1–14	911, 912	W78-W80	2,324	1,515	0.605	0.98

*RDS: Respiratory distress syndrome; BPD: Bronchopulmonary dysplasia; ASSB: Accidental suffocation and strangulation in bed; UNK: Unknown ill-defined unspecified causes; AULRI: Acute upper and lower respiratory infections; SIFFO: Suffocation from inhalation of food or foreign object; IGC: W78 Inhalation of gastric contents (not defined separately in ICD 9).

**Table 2:** Male mortality fractions of all applicable* 9ICD Chapters in US infants (<1 year) 1979–1998 with comparison to 10ICD equivalents for 1999–2005 [6].

Disease systems and disorders	9ICD** Chapter,1979–98	Male	Female	Male fraction	10 ICD** Chapter, 1999–05	Male	Female	Male fraction
Infectious and parasitic disease	001–139	8,518	6,705	0.560	A00-B99	2,069	1,652	0.556
Neoplasms	140–239	1,633	1,563	0.511***	C00-D48	482	472	0.505***
Endocrine, nutritional, metabolic diseases and immunity disorders	240–279	4,080	3,196	0.546	E00-E85	1,017	769	0.569
Blood and blood forming organs	280–289	959	773	0.554	D50-D89	385	260	0.597
Mental and behavioral disorders	290–319	115	112	0.507***	F01-F99	27	18	0.600
Nervous system and sense organs	320–389	7,542	5,792	0.566	G00-G98	1,556	1,147	0.576
Circulatory system	390–459	11,405	8,913	0.561	I00-I99	2,352	1,980	0.542
Respiratory system	460–519	14,313	10,055	0.587	J00-J98	2,725	1,959	0.581
Digestive system	520–579	4,843	3,385	0.588	K00-K92	2,249	1,494	0.601
Genitourinary system	580–629	2,629	1,966	0.572	N00-N98	762	551	0.580
Skin and subcutaneous tissue	680–709	81	71	0.533	L00-L98	14	1	0.933
Musculoskeletal system and connective tissue	710–739	121	94	0.563	M00-M99	60	24	0.714
Congenital anomalies—not respiratory system	740–747	73,154	64,609	0.540	Q00-Q29	18,173	16,643	0.522
	749–759				Q35-Q99			
Congenital anomalies—respiratory system	748	11,567	7,662	0.602	Q30-Q34	2,509	1,822	0.579
Certain conditions originating in perinatal period	760–779	198,282	151,191	0.567	P00-P96	55,803	42,795	0.566
Symptoms, signs, other unspecified conditions (includes SIDS 798.0 and R95)	780–799	67,116	44,603	0.601	R00-R99	14,435	9,557	0.592
External causes of injury and poisoning	800–999	13,970	11,187	0.555	V01-Y89	5,545	4,221	0.568

*9ICD codes 630–679 and 10ICD codes O00-O99 for pregnancy, childbirth, and puerperium complications are invalid for infants.

**The 9ICD codes provided for these general conditions may not match exactly to the 10ICD codes listed for the same general conditions.

***Male fraction is lower than the 0.512 male fraction of US live births expected if there is no male disadvantage.

**Table 3:** SIDS rate per 1000 live births increases with live-birth order, U.S. 1995–2004 [27] as compared to an infection vector model (*r* = 0.9966).

Live birth order	1	2	3	4	5	6 or more
SIDS rate per 1000 live births	0.491	0.691	0.816	1.033	1.199	1.294
Model Rate 2.5[1 − 0.9^(1+LBO)^]	0.475	0.678	0.860	1.023	1.171	1.304

**Table 4:** The −2*σ* lower limit of normal term infant Hemoglobin (Hb, g/dL) [33].

Age, weeks	0 (cord)	1	2	4	8	13–26	26–52
Hb g/dL (−2*σ*)	13.5	13.5	12.5	10	9	9.5	10.5
Deficit from 13.5 g/dL	0	0	1	3.5	4.5	4	3
